# Robust deepfake detector against deep image watermarking

**DOI:** 10.1371/journal.pone.0338778

**Published:** 2025-12-31

**Authors:** Jian Yu, Xin Liu, Fengbiao Zan, Yanhan Peng

**Affiliations:** School of Intelligence Science and Engineering, Qinghai Nationalities University, Xining, Qinghai, China; University of Baghdad, IRAQ

## Abstract

Deepfake technology poses a significant threat to information security,rendering deepfake detection research crucial. However, current detection methods experience a marked performance degradation in the presence of deep watermarking within images. In this paper, we propose a multi-module model, which integrates Efficient Multi-scale Attention within Xception as the detection module and introduces a feature dropout module to eliminate redundant image features. Experimental results demonstrate that when 50% and 100% of the images in the dataset contain MBRS watermarks, the accuracy (ACC) metrics of our model are comparable to those of existingbaseline models. However, when 50% and 100% of the images contain FaceSigns watermarks, the ACC metrics of our model outperform those of other baseline models by approximately 10% and 20%, respectively.

## Introduction

Recent advancements in deepfake technology have significantly enhanced the field of face synthesis. This progress is largely driven by breakthroughs in deep learning frameworks, such as generative adversarial networks (GANs) and variational autoencoders (VAEs). Currently, deepfake technology presents a substantial challenge to the credibility of social media content due to its low cost, ease of use, and high-quality generation capabilities. As a result, research aimed at countering deepfake technology has become critically important, with the identification of fabricated facial content online remaining a significant challenge.

Given the rapid advancement of deepfake technology, both academia and industry have actively pursued research on countermeasures, yielding several notable results. These efforts can be broadly categorized into three types: proactive forensics, passive forensics, and proactive defense techniques. Proactive forensics techniques involve the use of encoders to preprocess images initially. Following image manipulation, these techniques employ corresponding decoders to assess the authenticity of the images. Such approaches often rely on robust digital watermarking or steganographic embedding, such as reversible watermarking schemes resilient to geometric attacks [1], secret signal hiding based on color-space conversion [2], and semantic-aware protection methods guided by fine-grained human parsing in complex scenes [3]. Proactive defense methods focus on introducing adversarial perturbations to images, ensuring that, when manipulated, the images do not convey misleading information. At the video level, recent work has explored content-aware encryption of semantically critical segments using chaotic systems, thereby limiting the exploitable narrative structure of original footage [4]. Passive forensics leverages the robust feature-extracting capabilities of deep neural networks to learn the characteristics of forged images, facilitating their detection.

In the current domain of deepfake detection, passive forensics techniques do not require preprocessing of images and can directly detect manipulated content. These techniques have emerged as the primary method for identifying potentially forged content online, owing to their high adaptability and broad applicability. In contrast, some proactive forensics techniques employ deep learning-based digital watermarking, which involves embedding a specific watermark into images prior to their dissemination in order to protect particular objects. However, previous studies often treat these two techniques in isolation, overlooking the potential impact that deep watermarks in proactive forensics may have on the performance of passive detection algorithms. To address this issue, some researchers have proposed optimization modules to mitigate these negative effects [5]; however, their implementation necessitates modifications to pre-trained models. In contrast, optimizing the passive detection approach by refining training strategies allows a single model to adapt to image processing by various active defense forensics techniques without altering the model structure, thereby enhancing the overall system’s compatibility and generalization capabilities.

In this paper, we present a robust deepfake detection model that does not require prior knowledge of the data distribution containing target deep image watermark. During the training phase, the model is trained solely on the original dataset, while during the inference phase, it is tested on data embedded with deep image watermark to assess its performance. To evaluate the model’s robustness in predefined scenarios, we conduct multiple experiments using three major deepfake detection datasets. Under varying conditions of deep image watermarking distribution, we examine the model’s performance in scenarios where the data contain unknown deep image watermark. The contributions of this paper are as follows:

- We explore the innovative approach of discarding redundant image features during detection and introduce a feature dropout module, which enhances the model’s robustness against unknown deep image watermark noise. The module is designed with a simple five-layer architecture that includes downsampling, upsampling, and skip connections to eliminate features that interfere with classification.
- Leveraging existing deep image watermarking models, we construct a deep image watermarking pool module to simulate real-world deep image watermarked data and evaluate our model using watermarked images.
- To further improve robustness, we integrate an Efficient Multi-scale Attention mechanism into the classification model, thereby enhancing its capability to detect watermarked data.

## Related works

### Deepfake generation

In current research and practical applications, facial deepfake technologies can be broadly categorized into four types: facial manipulation, face swapping, attribute editing, and full-face synthesis. Among these, face swapping and facial manipulation typically require both source and target facial images, generating manipulated outputs based on the attributes of the source face. Face swapping techniques generally involve either cropping-and-pasting replacements [6] or face identity replacement [7], with the goal of forging the identity of the target face. Facial manipulation methods, on the other hand, transfer the movements and expressions of the source face to the target face [8]. Although all of these approaches produce synthetic facial content, attribute editing modifies features such as hair color, skin tone, age, and gender, and can also add accessories like glasses, using generative models [9,10]. The aforementioned methods rely on real reference data, whereas full-face synthesis generates entirely synthetic faces from random noise, without any reference input [11]. As forgery techniques continue to evolve and produce increasingly realistic content, the development of robust detection technologies has become imperative.

### Passive forensics

Leveraging the feature extraction capabilities of deep neural networks, contemporary deepfake detection methods typically build upon these architectures to identify forged characteristics within images and assess their authenticity. Zhang et al. [12] trained a model to detect sharp edges in the YCbCr color space for forgery identification. However, as deepfake generation techniques evolved, such overt artifacts became increasingly difficult to detect. Consequently, some researchers began treating forged images as originating from different distributions. For instance, Chen et al. [13] employed self-supervised learning to capture image consistency, while Wang et al. [14] utilized Transformer-based models to extract and compare local and global image features. Recognizing that social media compression severely degrades typical forgery traces, Liao et al. [15] proposed modeling facial muscle motion dynamics to detect deepfakes under low-quality conditions. Although these methods perform well within the same dataset, they often exhibit limited generalization across different datasets. To overcome this limitation, Shiohara et al. [16] introduced a self-mixing module to simulate forgery traces from multiple methods, and Liu et al. [17] developed a Fake Blender module based on self-mixed images. This module enhances the diversity of simulated forgeries and improves the generalization ability of detection algorithms.

### Proactive forensics

Digital image watermarking ensures the security of visual content by embedding and extracting information within images through algorithmic techniques, playing a critical role in digital media protection.Recent work has extended deep watermarking to handle real-world distortions such as screen shooting through grayscale deviation simulation [18] and wavelet-based recovery architectures [19]. At the same time, researchers have explored adaptive steganography using texture-aware payload allocation [20] and revealed new vulnerabilities via concealed adversarial attacks that impair watermark extraction while preserving visual fidelity [21]. Following the success of the HiDDeN model [22], which enabled end-to-end deep watermark generation, researchers have further investigated the broader applications of deep image watermarking. To address HiDDeN’s limitations under JPEG compression, Jia et al. [23] proposed the MBRS model, which was trained using real JPEG algorithms. Fang et al. [24] enhanced watermarking performance by jointly optimizing transparency and robustness. In the domain of deepfake defense, Beuve et al. [25] utilized semi-fragile watermarks for tamper detection, Zhao et al. [26] employed robust watermarks for identity verification, and Wu et al. [27] developed SepMark, a model capable of detecting watermarks across varying network depths. Unlike earlier methods that focused on protecting individual faces, FaceSigns by Neekhara et al. [28] introduced multi-target protection by embedding watermarks into multiple faces within a single image.

### Proactive defense

Similar to proactive forensics, proactive defense methods protect against deepfakes by introducing imperceptible image modifications that interfere with the generative process of deepfake models. One line of work aims to weaken the generation capabilities of manipulation systems. Yeh et al. [29] proposed the limit-aware self-guiding gradient sliding attack (LaS-GSA), which suppresses the generative ability of image-to-image translation GANs. He et al. [30] further used latent-space search to obtain images that remain robust under reconstruction, making them difficult to forge. Another line of research leverages adversarial perturbations for cross-model disruption. Huang et al. [31] introduced CMUA-Watermark by exploiting the transferability of adversarial examples, causing protected images to yield reconstructions with severe artifacts. More recently, Zhang et al. [32] proposed a unified framework that jointly embeds forensic watermarks and adversarial perturbations, providing both traceability and anti-forgery protection across diverse attack scenarios.

Previous research addressing deepfake countermeasures has predominantly focused on optimizing the performance of proactive forensics and passive forensics methods within self-defined scenarios. Passive forensics techniques have accounted for factors such as social media noise [33] and typical image quality degradation [34] during image transmission. Proactive forensics techniques, on the other hand, have considered challenges like image compression during distribution and the potential interference of watermarks with downstream tasks [5]. However, while proactive forensics methods may disrupt passive forensics, prior studies on passive forensics have seldom considered the impact of such interference from proactive forensics.

## Methodology

### Problem statement

Let ℰ and 𝒟 denote the encoder and decoder of the deep watermarking model, respectively, with *x* as the input image and *w* as the embedded information. Additionally, let ℱ represent the image similarity loss function. For instance, in the HiDDeN model, ℱ typically refers to the *l*_2_ loss used during training and optimization. Other models employ alternative loss functions, such as Mean Squared Error (MSE), Structural Similarity Index (SSIM), or Peak Signal-to-Noise Ratio (PSNR) loss. To enhance the realism of watermarked images, a discriminator assesses the output of the encoder, and its loss function, 𝒟, is commonly based on cross-entropy. ℬ represents the Bit Error Rate (BER) of the extracted watermarks. The global loss function of such models can be expressed by Eq (1), although the specific details vary across different algorithms.

$$\min_{ℰ,𝒟}{𝔼}_{\left(x,w\right)}[ℱ\left(ℰ(x,w),x\right)+𝒟\left(ℰ(x,w),y\right)+ℬ\left(𝒟\left(ℰ(x,w)\right),w\right)]$$ (1)

In images embedded with deep watermarking, the watermark information is inserted into the feature layers of the image, which are subsequently reconstructed into a watermarked image *x*_*w*_ through a deconvolution process. The embedded watermark can be approximately expressed as $w_{e}=x_{w}$ − *x*. Deep image watermarking typically does not produce a noticeable visual difference between the original and watermarked images. However, even minor alterations can affect the feature representations. As a result, conventional deepfake detection models may extract misleading features during the representation learning process, leading to erroneous predictions and a decline in detection accuracy.

### Deep image watermarking pool

**Fig 1:**
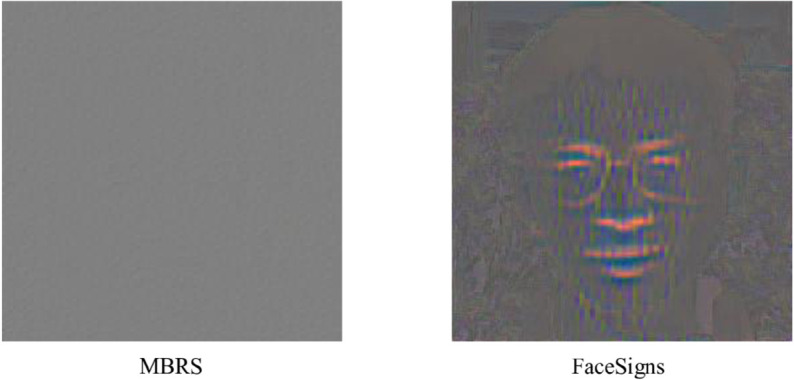
Comparison of spatial-domain watermark effects. Method *MBRS* yields an invisible watermark, whereas method *FaceSigns* introduces visible structural cues in the spatial domain.

Current deepfake detection datasets contain only original or forged images, without incorporating deep image watermark. However, since our model requires evaluation on watermarked data, environment simulation becomes essential. To facilitate this, we selected two models: MBRS, a general-purpose deep image watermarking model, and FaceSigns, a proactive forensic approach for deepfake detection. The watermarks embedded by FaceSigns remain intact even after processing by deepfake generation algorithms. After training both models, we integrated them into a unified deep image watermarking pool. This pool probabilistically adds watermarks to input images, where a proportion *p* determines that p×100% of the dataset images will be watermarked. During the watermarking process, the specific model used for embedding can be selected manually. Fig 1 illustrates the spatial-domain effects of watermarks added by the two methods. The watermark embedded by MBRS is nearly imperceptible in the spatial domain, whereas the watermark added by FaceSigns exhibits visible structural traces. Moreover, the module allows for embedding different types of watermarks across various samples in the dataset to enhance simulation fidelity. The watermark embedding process is defined by the Eq (2).

$$x^{\prime }=\left\{ \begin{array}{cc} x+w_{e}, & \text{if rand()}<p\\ x, & \text{otherwise} \end{array}\right.$$ (2)

### Model architecture

**Fig 2:**
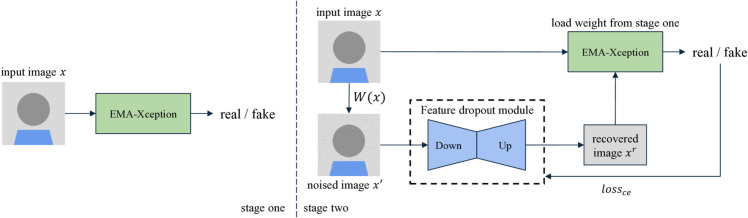
Overview of our proposed method. After two stages of training, our model maintains good performance regardless of whether the images contain watermarks or not.

As illustrated in Fig 2, the proposed model comprises two main components: the Feature Dropout (Fd) module and the classification module EMA-Xception. During the training phase, we first train the EMA-Xception network using the original dataset. Subsequently, to train the Fd module, we employ the watermark simulation module *W*(*x*) to simulate watermarked images. Afterward, the parameters of EMA-Xception are frozen, and its output is used to supervise the training of the Fd module. During inference, the model is evaluated on datasets processed by the deep image watermarking pool.

#### Feature dropout module.

**Fig 3:**
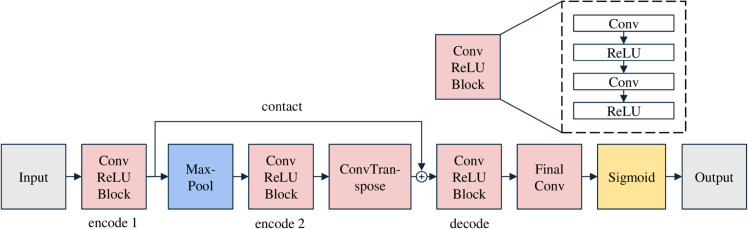
Architecture of the feature dropout module. This lightweight component consists of three core layers, including two encoding layers and one decoding layer. A skip connection is introduced between the first encoding layer (encode1) and the decoding layer (decode) to retain low-level features during feature suppression. The module processes input images while maintaining their spatial dimensions, ensuring that the output image size matches that of the input.

The architecture of the Fd module is illustrated in Fig 3. Most contemporary deep image watermarking models adopt an encoder-decoder framework, where watermark information is encoded as tensors and embedded into the image’s feature layers. The primary function of the Fd module is to eliminate redundant features from the original image, thereby reducing their interference with the classification model’s decision-making process. Similar to watermarking models, the module also employs an encoder-decoder structure. Following the U-Net design, a skip connection is incorporated between the encoder and decoder to facilitate information flow. Although fully convolutional networks and autoencoders are commonly used, deepfake detection tasks necessitate the retention of shallow features where forgery traces often reside. Conventional convolutional networks tend to discard these shallow features during processing; therefore, skip connections help preserve them, preventing the loss of critical forgery-related information.

#### EMA-Xception.

The Xception network [35] demonstrates strong performance in visual tasks such as image classification and semantic segmentation. Derived from the Inception architecture [36], it employs depthwise separable convolutions and cross-channel activation, along with global average pooling for feature aggregation. By replacing the standard Inception convolutions with depthwise separable convolutions, Xception introduces a novel modular structure that is both more efficient and computationally powerful. This design significantly reduces the number of parameters and model complexity while enhancing accuracy and generalization capabilities in visual recognition tasks.

However, the feature dropout module compresses images through convolutional operations and subsequently reconstructs them using deconvolution, which removes watermark features. Due to the irreversible nature of convolutional compression, features critical for determining image authenticity are also lost. Therefore, it is necessary to improve the multi-scale feature extraction capacity of the Xception backbone, integrate channel and spatial information, and enhance attention mechanisms. Since our task involves detecting unknown watermarked images, generalization is of paramount importance. To meet this requirement, we incorporated the Efficient Multi-scale Attention (EMA) [37] module into the Xception architecture, thereby improving its applicability to this task.

## Experiments

### Experiments settings

#### Dataset.

To evaluate the effectiveness of our method, we utilized widely recognized datasets commonly employed in deepfake detection: UADFV, CelebDF-v1 and CelebDF-v2. Although these datasets are publicly available, they necessitate preprocessing steps, including frame extraction, face cropping, and alignment. As a result, we opted for the preprocessed dataset from DeepfakeBench [38], which randomly selects 32 frames per video, aligns faces using facial landmark detection, crops them to a resolution of 256×256, and provides well-organized labels. Using subsets of DeepfakeBench as our baseline, we created four watermarked datasets with the deep image watermarking pool: 50% or 100% of the images are watermarked with MBRS, and 50% or 100% with FaceSigns.

#### Baseline.

**SRM** [39] extracts both frequency and spatial features using SRM filters and integrates them through cross-attention mechanisms to enhance the model’s generalization capability in forgery detection.

**UCF** [40] disentangles image information into three components: features unrelated to forgery, method-specific forgery cues, and common forgery patterns. It reconstructs images based on these forgery-related features and applies contrastive regularization to distinguish between different types of forgeries.

**CORE** [41] generates augmented views of input images, extracts features using a shared encoder, enforces representation similarity using cosine distance, and performs classification for each view using supervised labels.

**MINet** [42] first adaptively extracts several non-overlapping local features and uses mutual information theory to ensure they do not share redundant information. It then keeps task-relevant information, removes irrelevant details, and fuses all features into a compact global representation to determine whether the image is real or fake.

#### Implementation details.

**Fig 4:**
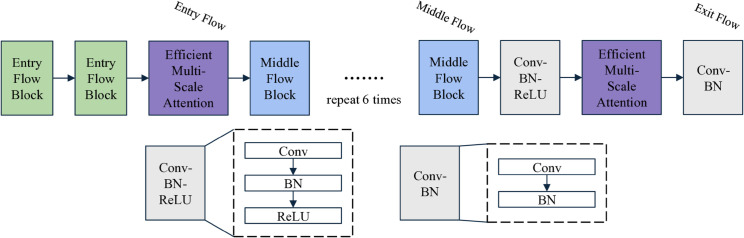
Architecture of EMA-Xception. A modified version of the Xception network with one EMA module integrated into both the Entry Flow and Exit Flow. The EMA modules are designed to capture multi-scale contextual features by aggregating information across different spatial resolutions, thereby enhancing the network’s capacity to model hierarchical visual patterns in the input (Entry Flow) and to refine global–local feature interactions in the output (Exit Flow).

**EMA-Xception.** The Middle Flow of the Xception architecture incorporates depthwise separable convolutions and residual connections, with these combined units organized into multiple stacked layers. This design reduces the model’s parameters and computational load while maintaining effective feature extraction. Building upon Xception, we introduced the EMA attention mechanism solely in the Entry and Exit Flows. In the Middle Flow, we stacked the convolution-residual units eight times. The updated model architecture is illustrated in Fig 4.

**Training.** The model training process is conducted in two stages. In the first stage, the classifier is trained on the original dataset images, and the optimization is performed using cross-entropy loss. In the second stage, the trained weights are loaded into the classifier, its parameters are frozen, and adversarial training is applied. Each batch consists of two categories of images: one real image from the dataset and one image processed by the frozen HiDDeN encoder and feature dropout module. The classifier compares these images to optimize the feature dropout module. Throughout the training process, only cross-entropy loss is used to maintain computational simplicity, and early stopping is employed to prevent overfitting.

### Experiment results

#### Problem verification experiment.

**Fig 5:**
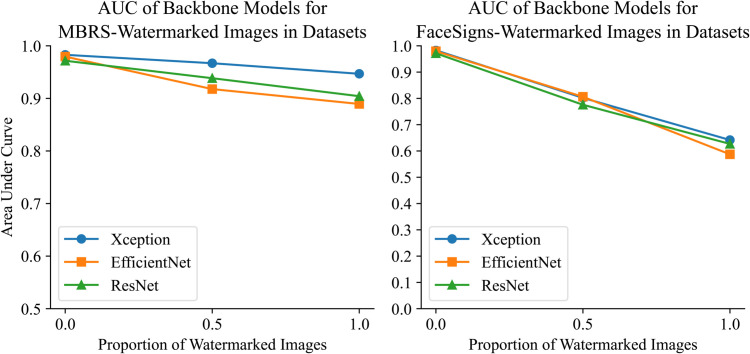
The result graph of problem verification experiment. Left panel: AUC scores of Xception, ResNet, and EfficientNet on datasets with increasing proportions of MBRS-watermarked samples (0, 0.5, 1). Right panel: Corresponding AUC scores for datasets watermarked using FaceSigns. Solid lines denote the mean values over three independent trials, while shaded regions represent the standard deviation. Both panels illustrate a consistent decline in model performance as the proportion of watermarked images increases.

In this section, we evaluate the impact of deep watermarking on detection models utilizing three commonly used backbone networks in deepfake detection: Xception, EfficientNet, and ResNet. All models were trained on the Celeb-V2 dataset. For robustness testing, 50% and 100% of the original Celeb-V2 images were replaced with watermarked versions. As shown in Fig 5, the performance of these backbone models declines when images with MBRS or FaceSigns watermarks are incorporated into the original dataset at varying ratios.

**Fig 6:**
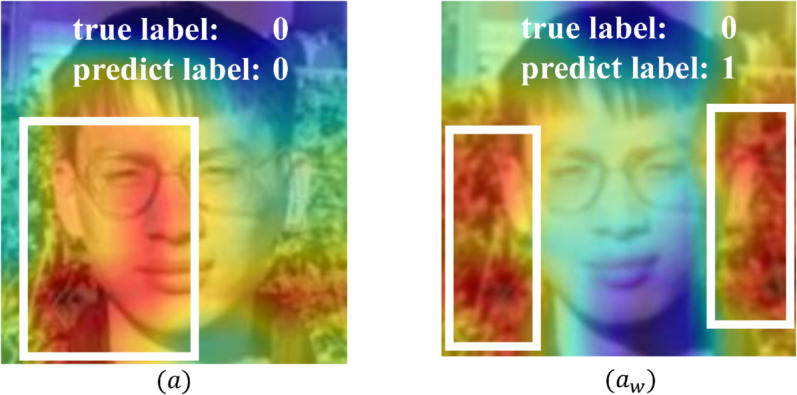
Grad-CAM visualizations of model attention for the original image (a) and the image embedded with a FaceSigns watermark (*a*_*w*_). Red regions indicate higher attention. White boxes are manually annotated to highlight the most concentrated attention areas for illustrative comparison. The observed shift in attention suggests that watermark embedding alters the model’s attention distribution.

To investigate the factors contributing to the decline in model performance, we utilized Gradient-weighted Class Activation Mapping (Grad-CAM) to analyze images that were correctly classified by the Xception model on the original dataset but misclassified after the introduction of FaceSigns watermarks. Heatmaps generated from the final convolutional layer of Xception were used to visualize the model’s attention regions, as shown in Fig 6. The results reveal significant differences in the model’s focus between the original and watermarked images, which ultimately led to misclassification errors.

#### Comparative experiment.

In this section, we conduct experiments on three mainstream datasets to fully assess the effectiveness of our proposed method for detecting images containing deep image watermarks. All models are trained using early stopping to prevent overfitting from affecting the results.

**Results on UADFV.** We conduct experiments on the UADFV dataset and compare the performance of our method against baseline models described in Section Baseline. As shown in Table 1, our model performs less effectively than the baselines on both the original dataset and the dataset embedded with MBRS watermarks. In contrast, our method outperforms all baseline models on the dataset containing FaceSigns watermarks. Furthermore, the consistent performance of our method across different datasets underscores its robustness to deep image watermark interference.

**Table 1:** Performance comparison of SRM, UCF, CORE, MINet, and the proposed method on the UADFV dataset, processed with FaceSigns and MBRS deep image watermarking models (AUC/ACC).

Methods	Origin	Deep Watermarking Method & Proportion				Average
		FaceSigns		MBRS		
		50%	100%	50%	100%	
SRM	**1.0/0.9984**	0.9694/0.8238	0.8722/0.6531	**0.9998**/**0.9845**	**0.9996**/0.9761	0.9682/0.8872
UCF	0.9991/0.9799	0.9531/0.8454	0.831/0.7057	0.999/0.9803	0.999/0.9806	0.9562/**0.8984**
CORE	0.9995/0.9816	0.9236/0.7812	0.7248/0.5811	0.9995/0.9815	0.9995/**0.9823**	0.9294/0.8615
MINet	0.9988/0.9832	0.9031/0.7861	0.6616/0.6034	0.9962/0.9535	0.9932/0.93	0.9106/0.8512
Ours	0.9845/0.8625	**0.9777**/**0.8931**	**0.9844**/**0.9161**	0.985/0.8693	0.986/0.8735	**0.9835**/0.8829

**Results on Celeb-DF-V1.** To assess the performance of our proposed method on the Celeb-DF-V1 dataset, we conduct experiments, with detection results presented in Table 2. The results show that our method surpasses other approaches in nearly all scenarios, clearly demonstrating its superior performance on Celeb-DF-V1.

**Table 2:** Performance comparison of SRM, UCF, CORE, MINet, and the proposed method on the Celeb-V1 dataset, processed with FaceSigns and MBRS deep image watermarking models (AUC/ACC).

Methods	Origin	Deep Watermarking Method & Proportion				Average
		FaceSigns		MBRS		
		50%	100%	50%	100%	
SRM	0.9899/0.9323	0.8186/0.6563	0.6402/0.3858	0.9399/0.8584	0.9337/0.7886	0.8645/0.7243
UCF	0.979/0.9311	0.8237/0.6531	0.6656/0.3836	0.9368/0.8817	0.8772/0.8351	0.8565/0.7369
CORE	**0.9957**/0.9404	0.7429/0.6588	0.4347/0.3841	0.9241/0.7589	0.885/0.5908	0.7965/ 0.6666
MINet	0.9891/0.9187	0.7973/0.6524	0.6067/0.3967	0.9404/0.9104	**0.9476**/0.9067	0.8562/ 0.757
Ours	0.9716/**0.941**	**0.89**/**0.8253**	**0.8132**/**0.7315**	**0.9482**/**0.9164**	0.944/**0.912**	0.9134/**0.8652**

**Results on Celeb-DF-V2.** Among the datasets utilized, Celeb-DF-V2 contains the largest volume of data, comprising 207,951 images. As shown in Table 3, the overall performance of our proposed method surpasses that of the comparative approaches. These results demonstrate that our method maintains strong detection performance even on large-scale datasets.

**Table 3:** Performance comparison of SRM, UCF, CORE, MINet, and the proposed method on the Celeb-V2 dataset, processed with FaceSigns and MBRS deep image watermarking models (AUC/ACC).

Methods	Origin	Deep Watermarking Method & Proportion				Average
		FaceSigns		MBRS		
		50%	100%	50%	100%	
SRM	**0.9848**/0.8929	0.7888/0.6223	0.6261/0.351	0.9381/0.8789	0.9402/0.8657	0.8556/0.7222
UCF	0.9806/**0.9155**	0.7538/0.6387	0.5745/0.3609	0.9476/**0.8963**	0.9449/0.8663	0.8403/0.7355
CORE	0.9797/0.8679	0.7906/0.6378	**0.678**/0.401	0.9534/0.8839	0.9594/**0.8987**	0.8722/0.7379
MINet	0.9792/0.8216	0.8041/0.746	0.6378/0.607	0.88/0.795	0.8189/0.768	0.824/0.7475
Ours	0.9838/0.9003	**0.848**/**0.7613**	0.629/**0.6398**	**0.9719**/0.8935	**0.9657**/0.8977	**0.8797**/**0.8185**

#### Ablation study.

To evaluate the effectiveness of the Fd and EMA modules under deep watermark interference, we train the model on Celeb-DF-V1 with all other experimental settings kept constant. We then conduct ablation studies using two deep watermark types (FaceSigns and MBRS) and two replacement ratios (50% and 100%), as shown in the Table 4.

**Table 4:** Ablation study of the Fd and EMA modules under deep watermark interference (51: with module, 55: without module).

Model	Fd	EMA	Origin	Deep Watermarking Method & Proportion				Average
				FaceSigns		MBRS		
				50%	100%	50%	100%	
a	✗	✗	**0.9828**	0.8019	0.6417	0.967	0.9468	0.868
b	✓	✗	0.9827	0.8604	0.7134	0.9097	0.904	0.874
c	✗	✓	0.967	0.8742	0.7524	0.9438	**0.9675**	0.901
base	✓	✓	0.9761	**0.89**	**0.8132**	**0.9482**	0.944	**0.9134**

The results show that using Fd or EMA alone can yield good performance in some settings, but their stability is limited. For example, the EMA module drops to an AUC of 0.64 when all data are embedded with FaceSigns deep watermarks. In contrast, the combined Fd + EMA (base) configuration remains consistently robust across all perturbations (AUC > 0.81) and achieves an average AUC of 0.9134 with no obvious weaknesses. This demonstrates that the two modules work synergistically to improve the model’s robustness and generalization against diverse deep watermarks.

A frequency-domain analysis further clarifies the role of Fd. Deep watermarks typically introduce redundant high-frequency patterns that can overwhelm subtle manipulative cues. The Fd module selectively suppresses these watermark-dominant components while largely preserving intrinsic forgery-related features. Quantitatively, averaged over the validation set, the low-, mid-, and high-frequency energies decrease from 41.31 to 32.33, 3.85 to 1.42, and 1.35 to 0.50, corresponding to suppression rates of 21.7%, 63.1%, and 62.8%, respectively. This asymmetric suppression explains why Fd effectively removes watermark redundancy without destroying the original forgery traces, supporting the observed improvement in detection robustness.

## Conclusion

Experimental results demonstrate that the addition of deep image watermarks to original images leads to a decline in detector performance. Existing deepfake detection models exhibit considerable performance degradation when processing images with unknown deep watermarks. To mitigate this issue, we propose a novel detection method that effectively counteracts watermark interference, without requiring watermarked data for training. Future research will focus on enhancing the model’s performance across diverse datasets while preserving its robustness.
